# Acquired von Willebrand syndrome (AVWS) type 2, characterized by decreased high molecular weight multimers, is common in children with severe pulmonary hypertension (PH)

**DOI:** 10.3389/fped.2022.1012738

**Published:** 2022-11-14

**Authors:** Ivonne Wieland, Franziska Diekmann, Julia Carlens, Laura Hinze, Katharina Lambeck, Thomas Jack, Georg Hansmann

**Affiliations:** ^1^ Department of Pediatric Hematology and Oncology, Hannover Medical School, Hannover, Germany; ^2^ Department of Pediatric Cardiology and Critical Care, Hannover Medical School, Hannover, Germany; ^3^ Department of Pediatric Pulmonology, Allergology, and Neonatology, Hannover Medical School, Hannover, Germany

**Keywords:** acquired von willebrand syndrome (AVWS), pulmonary hypertension, bleeding risk, high molecular weight multimers, children

## Abstract

**Background and objectives:**

Emerging evidence suggests that increased degradation of von Willebrand factor and decrease in high molecular weight multimers occurs in patients with pulmonary hypertension (PH). However, the link between acquired von Willebrand Syndrome (AVWS) type 2 and PH remains poorly understood.

**Material and methods:**

We retrospectively evaluated the charts of 20 children with PH who underwent bilateral lung transplantation (LuTx) between 2013 and 2022. Von Willebrand variables were determined in 14 of these patients; 11 patients had complete diagnostics including multimer analysis.

**Results:**

We confirmed AVWS in 82% of the children studied (9 of 11 patients by multimer analysis). The two remaining patients had suspected AVWS type 2 because of a VWF:Ac/VWF:Ag ratio of <0.7. Platelet dysfunction or suspicion of VWD type 1 were found in two separate patients. All but one of the 14 children with severe PH had a coagulation disorder. Most patients (9 proven, 2 suspected) had AVWS type 2. Notably, 3 of 5 patients (60%) with normal VWF:Ac/VWF:Ag ratio >0.7 had abnormal VWF multimers, indicating AVWS type 2. Hemostatic complications were observed in 4 of 12 (33%) patients with VWS and 3 of 6 (50%) patients without diagnostics and therapy.

**Conclusion:**

For children with moderate to severe PH, we recommend systematic analysis of von Willebrand variables, including multimer analysis, PFA-100 and platelet function testing. Awareness of the diagnosis “AVWS” and adequate therapy may help to prevent these patients from bleeding complications in case of surgical interventions or trauma.

## Introduction

Von Willebrand factor (VWF) is a protein, which is required for adhesion, playing an important role in hemostasis. Various hereditary types (type 1–3) of von Willebrand disease (VWD) are described, in which mainly a reduction of VWF and/or VWF-multimers is involved (1). According to published data on the prevalence of VWD, hereditary VWD is found in up to 1% of the population, but the proportion of patients with VWD with clinical relevance is even smaller (one in 10,000 people) (1). VWD type 1 with reduced VWF, is the most common type (70%–80%), followed by type 2 that is characterised by reduction or loss of large VWF multimers (20%–25%), and type 3 with a complete loss of VWF (<1%–5%) (1, 2). Acquired von Willebrand Syndrome (AVWS) was reported to have a prevalence of 0.04% to 0.13% in the population (3). Generally, AVWS is associated with an underlying disorder. According to the International Society on Thrombosis and Haemostasis (ISTH) registry (4) and a recent report (5), the most common conditions associated with AVWS are: lymphoproliferative/haematological malignancy (48%), cardiovascular (21%), myeloproliferative (15%), other neoplastic (5%) and autoimmune disorders (2%–5%) or various other causes (plasma-mediated hyperfibrinolysis, glycogen storage disease, uremia, hypothyroidism) (<10%) (4, 5).

In the paediatric population, AVWS tends to be underdiagnosed and often unknown. However, data on AVWS in childhood are rare and mostly case reports or small case series. Most data are reported for AVWS in pediatric patients with congenital heart diseases (6–14).

Acquired AVWS as type 2A is especially common in adult patients with aortic valve stenosis (vAS) (15). 67%–92% of patients with severe aortic stenosis (vAS) are reported to develop AVWS, and 21% of those patients suffer from bleedings (16). Pathophysiologically, it was assumed that the development of AVWS in vAS is caused by the acceleration of blood flow at the aortic valve, resulting in shear stress for large plasma proteins like VWF, leading to consecutive proteolytic cleavage of VWF and a decrease or loss of the high molecular weight multimers (HMWMs). In addition to aortic stenosis, AVWS was reported for instance in hypertrophic obstructive cardiomyopathy (HOCM), tetralogy of Fallot, pulmonary hypertension and mitral regurgitation (17).

According to the World Symposium on Pulmonary Hypertension (WSPH, 2018), pulmonary hypertension (PH) is a condition that is divided into 5 subgroups (18–20).

The pathobiology of pulmonary arterial hypertension (PAH) is a complex and multifactorial process, in which peripheral artery loss and obstructive vascular remodeling cause a rise in pulmonary arterial pressure (PAP) and pulmonary vascular resistance (PVR), resulting in progressive right heart failure and death (21). Inflammation, delayed shear adaptation and endothelial cell dysfunction seem to play crucial roles in this process (22–25). Wall shear stress-dependent changes in pulmonary arterial lumen diameter were found to be a persistent remodeling response (26).

Especially in young patients, the etiology of PH (groups 1–5) is very heterogeneous across the different age groups, but most frequently associated with congenital heart disease (CHD; group 1 PH = pulmonary arterial hypertension), followed by developmental lung disease (group 3 PH; mainly bronchopulmonary dysplasia) and so-called idiopathic PAH forms (18, 19, 21). The estimated incidence for idiopathic PAH (IPAH)/heritable PAH (HPAH) and (non-transient) CHD-associated PAH is 0.7 and 2.2/million, and the estimated prevalence is 4.4 and 15.6/million children, respectively. (19).

From the late 1980s, emerging evidence suggested that PAH patients have increased degradation of VWF and a decrease in high molecular weight multimers, and/or platelet dysfunction, which are typical findings of AVWS type 2 (27). Abnormal flow or shear stress through the pulmonary vessels has been suggested to cause PAH in 1995 (28). A study on 30 PAH patients showed that those patients with abnormalities in VWF had a reduced 1-year survival rate compared with those with normal VWF (29).

Apart from isolated cases, such as the case report on an adolescent woman with PAH and menorrhagia suffering from AVWS type 2 (30), very little data on AVWS in pediatric PH has been published so far. In another cohort of 16 patients with CHD, 5 had PAH. All of them suffered from AVWS type 2 and presented with bleeding symptoms such as epistaxis, menorrhagia or gum bleeding (31). Surprisingly, in a small study on 8 children with PAH, all of them had AVWS type 1 with a normal multimer analysis differing from other reports describing an association with abnormalities in VWF or AVWS type 2 (32).

In this retrospective study, we analyzed the von Willebrand variables in all children with severe PH and right ventricular (RV) failure undergoing lung transplantation (LuTx) at Hannover Medical School between December 2013 and February 2022. We hypothesized that most end-stage PH patients have evident AVWS type 2 that is relevant to patient management perioperatively (e.g., VWF supplementation).

## Material and methods

**Figure 1:**
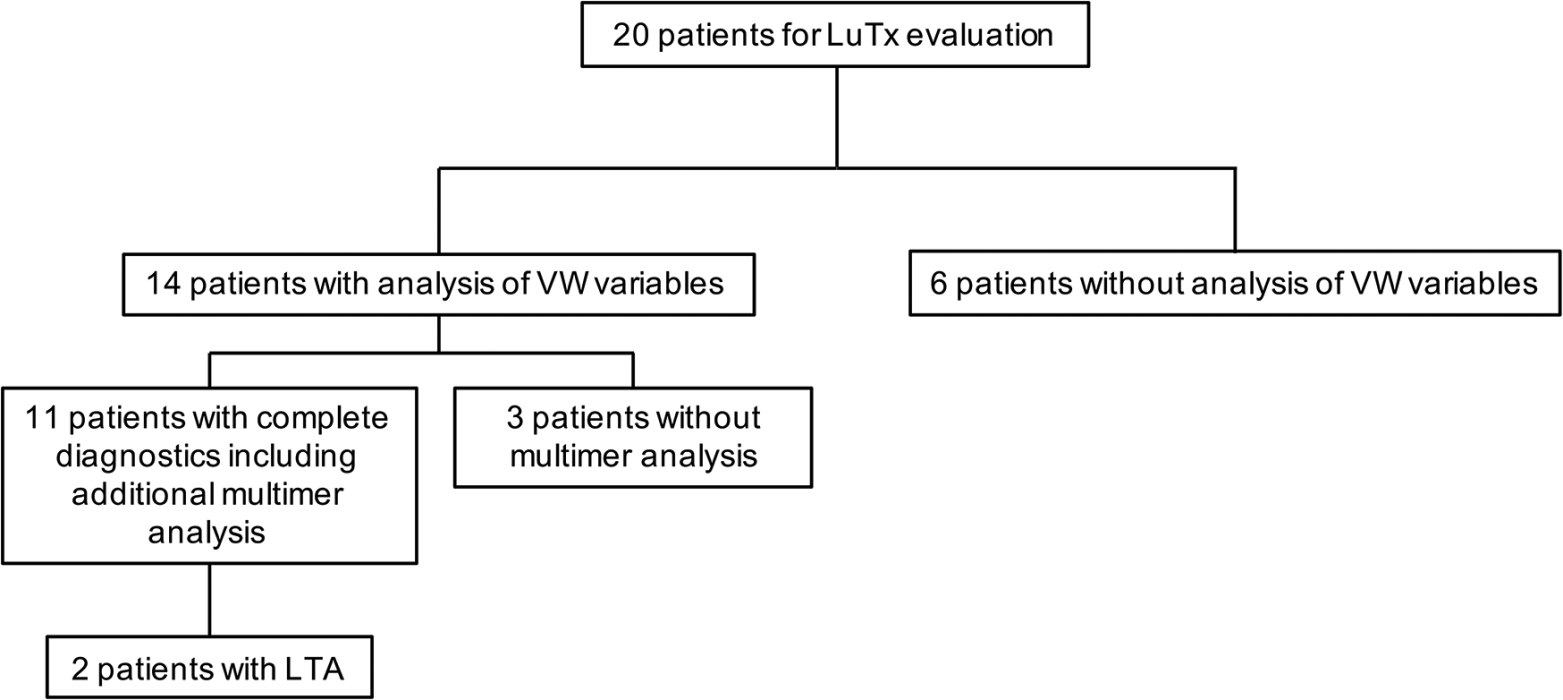
Diagnostic workup. The indicated VW variables include von Willebrand factor activity (VWF:Ac) and von Willebrand factor antigen (VWF:Ag). LTA, light transmission aggregometry; LuTx, lung transplantation; VW, von Willebrand.

We retrospectively evaluated the charts of 20 children with PH undergoing lung transplantation at Hannover Medical School between December 2013 and February 2022 for von Willebrand variables (von Willebrand factor activity (VWF:Ac); von Willebrand factor antigen (VWF:Ag), multimers) and, when performed, for platelet function analysis (Figure 1).

Measurement of von Willebrand parameters were performed once during evaluation for lung transplantation in the central laboratory of MHH. VWF:Ag and VWF:Ac were determined in the Siemens instruments tubimetricly. PFA-100 was measured on COLL/EPI and COLL/ADP membranes on a Siemens instrument. For platelet function analysis, light transmission aggregometry (LTA, so called “Born-Aggregation”) was performed in the APACP 4S Plus/DiaSys instrument.

Von Willebrand Multimer analysis was done externally by the laboratory AMEDES, Hamburg, following standardized protocols.

Patients with a VWF:Ac/VWF:Ag ratio <0.7 are suspected of having VWS type 2 according to the ASH guidelines (33). AVWS type 2 was confirmed with a loss of largest and reduction of large multimers in multimer analysis independent of the VWF:Ac/VWF:Ag ratio.

Von Willebrand variables (VWF:Ag and/or VWF:Ac) ≤ 50% were classified as suspected VWD type 1. We cannot differ between congenital or acquired.

Von Willebrand variables, at least in our laboratory, were determined in only 14 of the 20 patients. Twelve of these 14 patients were female. The mean age at transplantation was 10.9 years (range 1.9–21.3 years) (Table 1, Supplementary Table S1).

**Table 1:** Characteristics of the 20 PH patients studied.

Patients #1–20	PH patients *N* = 20
**Demographics**	
Age – years (range)	10.9 ± 1.2 (1.9–21.3)
Sex, Female – *n* (%)	16 (80%)
Height – m	1.4 ± 0.1
Weight – kg	32.3 ± 3.8
BSA – m^2^	1.1 ± 0.1
**Clinical Diagnosis**	
PH Group 1 − *n*	19
1.1 IPAH	8
1.2 HPAH (BMPR2, *n* = 4; TBX4, *n* = 1)	5
1.4.4 PAH-CHD	3
1.6 PVOD/PCH	3
PH Group 3 − *n*	1
WHO Functional Class	3.6 ± 0.1
NTproBNP – ng/L	3065.9 ± 814.8
**Invasive Hemodynamics**	
mRAP – mm Hg, *n* = 16	8.1 ± 1.0
RVEDP – mm Hg, *n* = 15	12.3 ± 0.7
mPAP – mmHg, *n* = 17	74.3 ± 4.2
mPAP/mSAP, *n* = 16	1.1 ± 0.0
PVRi – WU·m^2^, *n* = 17	24.0 ± 2.4
PVR/SVR, *n* = 17	1.3 ± 0.1
Qsi – L/min/m^2^, *n* = 16	3.6 ± 0.5

Values are presented as mean ± SEM. If the patient has a mutation that is associated with PAH, he/she belongs to group 1.2 PH (HPAH). One patient (listed here as IPAH) may also be classified as PAH-CHD (group 1.4.4). The serum N-terminal prohormone of brain natriuretic peptide (NTproBNP) concentrations are the last measurements prior to lung transplantation (LuTx). Only catheterization data from the previous 12 months prior to LuTx are shown. One patient did not undergo catheterization at all and two patients did not have a cardiac cath within the 12 months before LuTx. BSA, body surface area; cath, catheterization; CHD, congenital heart disease; HHT, hereditary hemorrhagic telangiectasia; HPAH, hereditary PAH; IPAH, idiopathic PAH; LuTx, lung transplantation; mPAP, mean pulmonary arterial pressure; mRAP, mean right atrial pressure; mSAP, mean systemic arterial pressure; NT-proBNP, N-terminal pro b-type natriuretic peptide; PAH, pulmonary arterial hypertension; PCH, pulmonary capillary hemangiomatosis; PVOD, pulmonary venoocclusive disease; PVR, pulmonary vascular resistance; PVRi, pulmonary vascular resistance index; Qsi, systemic flow index; RVEDP, right ventricular end-diastolic pressure; SVR, systemic vascular resistance; WHO, World Health Organization.

Nineteen patients were classified as group 1 PH (idiopathic (*n* = 8) or heritable PAH (*n* = 5), PAH-CHD (PAH associated with congenital heart disease, n = 3) and PVOD/PCH [pulmonary veno-occlusive disease PVOD)/pulmonary capillary hemangiomatosis (PCH), n = 3] and one as group 3 PH (PH associated with developmental lung disease) (Table 1, Supplementary Table S1).

All patients underwent ECMO peri transplantation. The management of PAH patients undergoing LuTx at our center using veno-arterial ECMO (VA-ECMO) support has previously been published (34, 35). Only patients with known or suspected VWS received VWF supplementation during VA-ECMO support pre and post LuTx, no patient without known VWS. We further identified complications related to hemorrhage and thromboembolic events in all 20 pediatric patients pre and post lung transplantation (LuTx).

## Results

### Analysis of von Willebrand variables and platelet function

Von Willebrand variables (VWF:Activity (VWF:Ac) and VWF:Antigen (VWF:Ag)) in our locale laboratory were determined in 14 of these patients, but the diagnostic workup was complete in only 11 patients and confirmed by multimer analysis (Figure 1).

**Figure 2:**
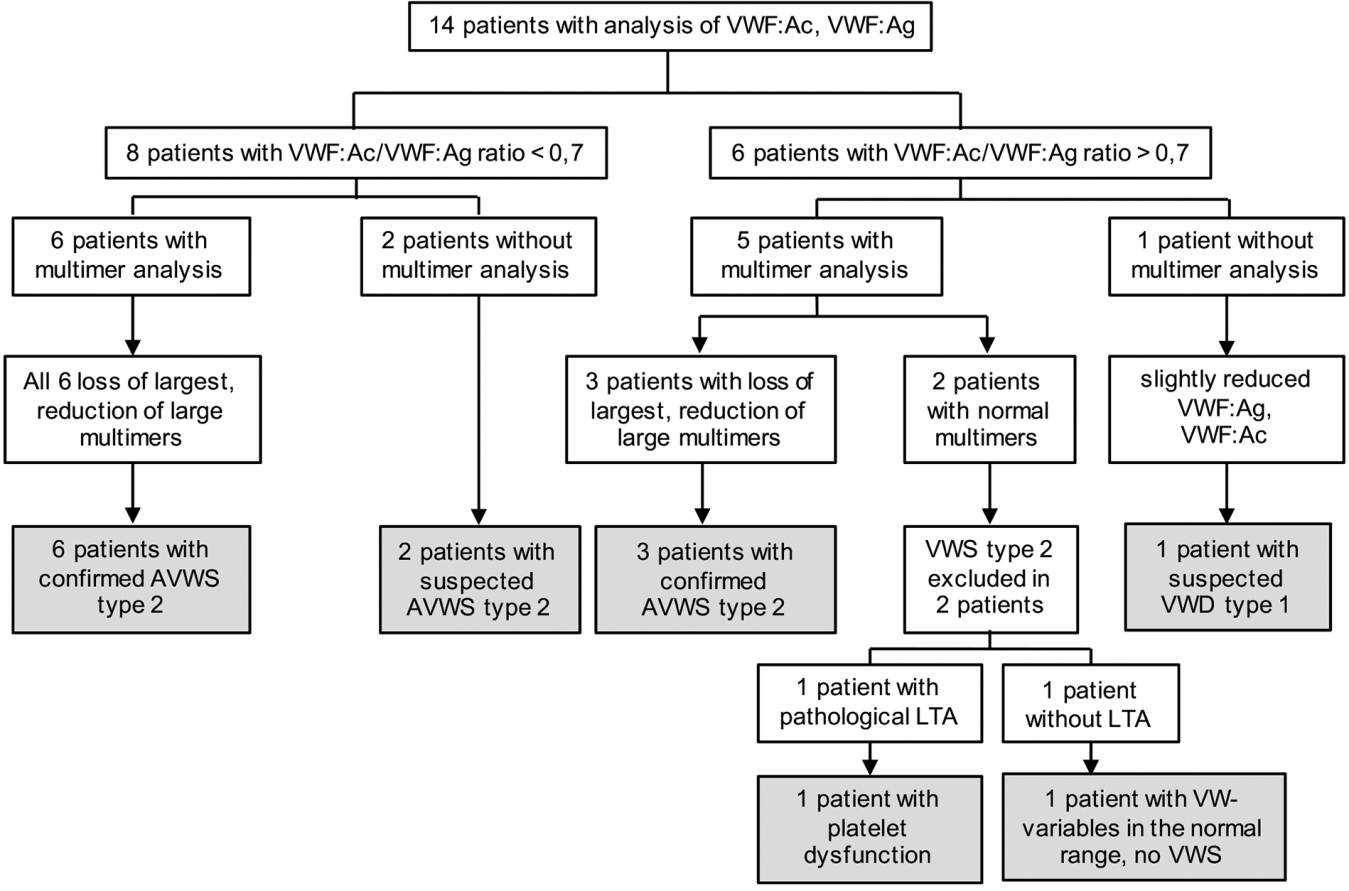
Results of diagnostic workup. AVWS, acquired von Willebrand syndrome; LTA, light transmission aggregometry, VWF: Ac, von Willebrand factor activity; VWF: Ag, von Willebrand factor antigen; VWS, von Willebrand syndrome.

In these 14 patients, we could find 9 (64%) with confirmed AVWS type 2, 2 (14%) with suspected AVWS type 2, 1 (7%) platelet dysfunction and 1 (7%) with suspected VWD type 1 (without excluded type 2 because of missing multimer analysis) (Figure 2, Table 2).

**Table 2:** Results: laboratory results and hemostaseological diagnosis.

ID	VW-Variables before LuTx					Platelet function	Diagnosis
	VWF: Ac	VWF: Ag	Ratio Ac/Ag	PFA-100	Multimer analysis	LTA	
2	139.8	260	0.54	N/A	N/A	N/A	**Suspected AVWS type 2**
4	98.7	149	0.66	N/A	Relative reduction of large multimers	N/A	**AVWS type 2**
7	49.7	73	0.68	N/A	N/A	N/A	**Suspected AVWS type 2**
8	51.5	46	1.1	N/A	N/A	N/A	**suspected VWD type 1**
9	129	80	1,6	>300	Loss of largest, reduction of large multimers	Pathological	**AVWS type 2**
10	63	46	1.4	N/A	Loss of largest, reduction of large multimers	N/A	**AVWS type 2**
11	62	71	0.87	>300	*Normal*	Pathological	**platelet dysfunction**
12	99	151	0.63	N/A	Loss of largest, reduction of large multimers	N/A	**AVWS type 2**
14	32	54	0.59	N/A	Loss of largest, reduction of large multimers	N/A	**AVWS type 2**
15	37.4	58.6	0.63	N/A	Loss of largest, reduction of large multimers	N/A	**AVWS type 2**
16	29	37	0.78	>300	Loss of largest, reduction of large multimers	N/A	**AVWS type 2**
17	62.6	79.7	0.78	N/A	Loss of largest, reduction of large multimers	N/A	**AVWS type 2**
19	53.8	77.2	0.69	N/A	Loss of largest, reduction of large multimers	N/A	**AVWS type 2**
20	83.7	87.4	1	N/A	*Normal*	N/A	No VWS

AVWS, acquired von Willebrand syndrome; N/A, not available; PFA-100, platelet function analyzer; VWF: Ac, von Willebrand factor activity; VWF: Ag, von Willebrand factor antigen; VWS, von Willebrand syndrome.

Eight of the 14 patients (57%) were suspected for AVWS type 2 due to a ratio of <0.7. In six of these patients, multimer analysis was performed and all of them (100%) demonstrated a decrease or loss of HMWM, which is typical of AVWS type 2. The remaining two patients were classified as suspected AVWS type 2 because of missing multimer analysis.

Six of the 14 patients (43%) had a normal VWF:Ac/VWF:Ag ratio (>0.7). In five of them, multimer analysis was performed. Despite a normal VWF:Ac/VWF:Ag ratio of >0.7, 3 of these 5 patients (60%) had a decrease or loss of HMWM, indicating these patients suffer from AVWS type 2. In 2 of these 3 patients, PFA-100 was performed and was highly increased (>300s).

Of the 2 patients with excluded AVWS type 2 by multimer analysis, one patient (patient 11) had pathological results in platelet function analysis. Despite normal platelet numbers, PFA-100 was highly increased (>300s) and in LTA, aggregations of each stimulus (ADP 10 µM, adrenalin 5 µM, collagen 5 µg/ml and ristocetin 1.0 mg/ml) were decreased, indicating platelet dysfunction in this patient. Due to the slightly reduced von Willebrand variables, patient 8 is suspected for at least VWD type 1, without fully excluded type 2 because of missing multimer analysis.

### Bleeding complications and thromboembolic events peri-LuTx

Four patients were bridged to transplantation on veno-arterial ECMO. All patients were transplanted on cardiopulmonary bypass or ECMO and all but one (patient number 6) remained on planned ECMO support after transplantation. Patients on ECMO and/or cardiopulmonary bypass received anticoagulation. Patients with known or suspected VWS were treated preventively with VWF containing concentrate during VA-ECMO to prevent bleeding complications.

We observed bleeding complications (hematothorax) in 4 patients (patients 5, 16, 18, 19) in the first days after transplantation. Two (2 of 12; 17%) of these occurred in patients with confirmed/suspected AVWS and two (2 of 6; 33%) in patients without extended hemostatic diagnostic work-up. One additional patient (number 1) suffered from hematothorax because of cannula dislocation during emergency ECMO cannulation before transplantation. Because the bleeding was caused by cannula dislocation, we do not include this patient in the group with bleeding complications.

We observed thromboembolic complications in 6 patients (patients 2, 5, 12, 13, 16, 18): four developed emboli after lung transplantation with ischemia in limb arteries during ECMO therapy or shortly after ECMO explantation, one suffered an infarction of the A. cerebri media on ECMO and one suffered a spinal cord ischemia before lung transplantation while on bridge-to-transplantation ECMO support. Three (3 of 12; 25%) of these occurred in patients with confirmed/suspected AVWS and three (3 of 6; 50%) in patients without extended hemostatic diagnostic work-up.

Both bleeding and thromboembolic events occurred in 3 patients [1 with AVWS (patient 16) and 2 without diagnostic workup (patients 5, 18)]. Details are summarized in Table 3.

**Table 3:** Diagnosis and hemostaseologic complications peri- translantation.

ID	Diagnosis	Hemostaseological Complications peri-transplantation
1	N/A	**Hematothorax** **due to cannula dislocation** during emergency ECMO cannulation before LuTx listing
3	N/A	None
5	N/A	**Hematothorax** (after central ECMO cannulation) after transplantation, **Stroke** of A. cerebri media post LuTx on ECMO
6	N/A	None
13	N/A	**Embolus and leg ischemia** after ECMO explantation requiring second embolectomy post LuTx
18	N/A	**Hematothorax after transplantation;** **spinal cord ischemia** with bilateral leg paralysis on pre-LuTx ECMO
20	No VWS	None
11	Platelet dysfunction	None
2	**Suspected AVWS type 2**	**Embolus** in right A. iliaca post LuTx after ECMO
4	**AVWS type 2**	None
7	**Suspected AVWS type 2**	None
8	**Suspected VWD type 1**	None
9	**AVWS type 2**	None
10	**AVWS type 2**	None
12	**AVWS type 2**	**Embolus** in right A. brachialis post LuTx after ECMO
14	**AVWS type 2**	None
15	**AVWS type 2**	None
16	**AVWS type 2**	**Hematothorax after transplantation**; **Embolisms** Aa femoralis, radialis and ulnaris left after ECMO explantation requiring second embolectomy, Pulmonary embolism post LuTx.
17	**AVWS type 2**	None
19	**AVWS type 2**	**Bilateral hematothorax** post LuTx on ECMO

N/A, not available; AVWS, acquired von Willebrand syndrome; VWS, von Willebrand syndrome, ECMO, extracorporal membrane oxygenation. A, arteria.

## Discussion

The detection of potential bleeding disorders is important in patients with advanced cardiovascular and pulmonary disease, including group 1 PH (PAH) and group 3 PH, especially if those patients undergo major surgery such as lung transplantation or creation of endogenous Potts shunt (35–37).

In all but one of the 14 children with severe PH from our cohort with diagnostics, we detected a coagulation disorder. AVWS type 2 (confirmed or suspected: 11/13; 85%) was the most common coagulation disorder. We found confirmed AVWS in 9 of 14 (64%) patients [9 of 11 children with complete workup (82%)]. Two more patients were suspected for AVWS type 2 because of VWF:Ac/VWF:Ag ratio <0.7. Two others suffered from platelet dysfunction (n = 1) or were suspected of having at least VWS type 1 (n = 1; without fully excluded type 2 because of missing multimer analysis). In one patient without coagulation abnormality, platelet function analysis was missing.

Multimer analysis is proving AVWS type 2 and should be performed in all patients if AVWS type 2 is suspected. However, multimer analysis is a time-consuming method only available in some specialized laboratories. For urgent clinical questions other tests are required that initially indicate the presence of AVWS type 2 - the VWF:Ac/VWF:Ag ratio <0.7 (33). In all our patients with a VWF:Ac/VWF:Ag ratio <0.7, multimer analysis confimed AVWS type 2. However, 60% of the patients with a normal VWF:Ac/VWF:Ag ratio >0.7 also had a decrease or loss of HMWM, indicating these patients suffer from AVWS type 2 too. This finding is in line with other reports (13, 38). For instance, Icheva et al. reported for their cohort of patients with CHD a very high specificity (100%) of the ratio VWF:RCo/VWF:Ag (RCo; Ristocetin-Cofactor) to detect an AVWS, but a very low sensitivity (38%). (13). In contrast, Tiede et al. found a sensitivity of 86% (38). Because of the relatively low sensitivity of the VWF:Ac/VWF:Ag ratio, some patients with AVWS type 2 can be missed using only this method. Possibly, this diagnostic uncertainty could be reduced by additional analysis of PFA-100, for which a sensitivity of >90% has been described for VWD type 2 (39). Concerning the sensitivity and specificity of the different tests, further investigations are necessary.

The pathophysiology of AVWS in PH seems to be comparable to that in patients with aortic stenosis and may also result from increased shear stress for large plasma proteins like VWF. The latter leads to consecutive proteolytic cleavage of VWF and a decrease or loss of HMWMs (24).

We found a higher proportion of patients with AVWS type 2 in our pediatric cohort with end-stage PH than previously reported (32). In the small study of Pelland-Marcotte, 8 of 14 patients with PAH have shown bleeding symptoms and/or laboratory abnormalities but with normal VW-multimers, surprisingly excluding type 2 AVWS. One of these patients was in NYHA functional class (FC) III and underwent lung transplantation. The 7 other patients were in NYHA FC I (*n* = 4), NYHA FC I-II (*n* = 2) and NYHA II-III (*n* = 1). In contrast, we only analyzed patients with severe PAH immediately prior to LuTx. Therefore, the results of our study may indicate that the risk of AVWS increases with the severity of PAH. This observation is in line with the much earlier report of Lopes et al., demonstrating that patients with PAH and abnormalities in circulating VWF have a reduced 1-year-survival (29).

The clinical importance of an increased bleeding risk in patients with AVWS is the subject of a controversial discussion (15, 40–42). On the one hand, published data show that AVWS is not or only mildly associated with a relevant bleeding risk in adults with aortic stenosis (16). No increased bleeding tendency was reported, neither in daily life nor during surgery of aortic valve stenosis in adults (40–42). On the other hand, a meta-analysis of patients with AVWS, including data from two different registries, demonstrated an increased rate of patients (77%) with bleeding diathesis in the subgroup of patients with underlying cardiovascular disease (4). However, data on the prevalence and clinical bleeding tendency in PH patients with AVWS, especially in children with PH, are missing. Recently, a report on a patient with end-stage PAH and a major bleeding following reverse Potts shunt procedure was published (43). Two other small clinical studies showed that 4 of 5 patients with PAH (31) and 7 of 8 patients with PH (32) suffered from bleeding symptoms (31, 32), suggesting a high percentage of bleeding in VWS associated with PAH. The most common bleeding problems included epistaxis, menorrhagia, and perioperative bleedings like hereditary VWD (30–32, 43).

Re-Thoracotomy for major bleeding (hematothorax) is a major and common complication in the first hours and days after lung transplantation in young patients with severe PAH. In 117 lung transplanted pediatric patients from our center (including children with pulmonary hypertension and other diagnoses), 13 (11.1%) required re-thoracotomy for hematothorax after transplantation (35). Based on our findings and previous experience (35, 36), we substitute VWF containing concentrate in all PAH patients with confirmed or suspected VWS type 1 or 2, prior to, during and after invasive procedures or surgery, and also during VA-ECMO while being heparinized.

In the group of patients with VWS, 4 of 12 patients (33%) suffered from hemostatic complications (2 embolisms, 1 hematothorax, 1 both). In the group without extended diagnostics, three of six patients (50%) had hemostatic complications (1 thromboembolism and 2 both, thromboembolisms and hematothoraces). These complications were usually in temporal relation to the ECMO support. The etiology of these complications is likely multifactorial. Patients on VA-ECMO support require anticoagulation with heparin but were all substituted with coagulative agents at the same time. Both bleedings and thrombotic events are well known complications in ECMO treatment. Despite substitution with VWF containing concentrate to improve hemostasis, we did not observe an increase of thromboembolic events in the group with VWS (25% vs. 50%). Due to the small number of patients, a statistic analysis between AVWS and clinical outcome was not possible. Further investigations are necessary to determine whether hemostatic diagnostics can improve clinical outcome.

The limitation of our study is the relatively small number of patients. However, these patients showed a low heterogeneity and a comparable disease severity at the time of analysis shortly before LuTx.

## Conclusion

Due to the high risk of bleeding complications in patients with severe PH, we recommend analysis of von Willebrand variables (VWF:Ag, VWF:Ac), multimer analysis, PFA-100 and platelet function testing [light transmission aggregometry (LTA, “Born-Aggregation”)] in all patients with severe PH. At least, multimer analysis is evidential for AVWS type 2 and should be performed in all of these patients. Early suspicion of evident AVWS type 2, before receiving the result of the mandatory multimer analysis, should be raised in case of a VWF:Ac/VWF:Ag ratio <0.7 and a prolongation of PFA-100. Of note, because of relatively low sensitivity of this ratio, some patients with AVWS could be missed. The recommendation to use the VWF:Ac/VWF:Ag ratio is for practical reasons, because multimer analysis is a time-consuming method and not available in most hospitals.

Early diagnosis of AVWS type 2 in critically ill PH patients undergoing ECMO cannulation and/or major surgery could give the health care providers the opportunity to treat or prevent potential hemorrhagic events and may improve the patients' safety and outcomes.

## Data Availability

The original contributions presented in the study are included in the article/Supplementary Material, further inquiries can be directed to the corresponding author/s.
