# Supplementary material for: Acquired von Willebrand syndrome (AVWS) type 2, characterized by decreased high molecular weight multimers, is common in children with severe pulmonary hypertension (PH)
Source: Front Pediatr. 2022 Nov 14;10:1012738. doi: 10.3389/fped.2022.1012738 (PMC9701817; doi:10.3389/fped.2022.1012738)
Supplement: Supplementary file 1 [file Datasheet1.docx]

**Supplementary Material**

**Acquired von Willebrand Syndrome (AVWS) Type 2, characterized by decreased high molecular weight multimers, is common in children with severe pulmonary hypertension (PH)**

Ivonne Wieland^1*^, Franziska Diekmann^2^, Julia Calens^3^**,** Laura Hinze^1^, Katharina Lambeck^1^, Georg Hansmann^2*^

**Author affiliations:**

^1^ Department of Pediatric Hematology and Oncology, Hannover Medical School, Germany

^2^ Department of Pediatric Cardiology and Critrical Care, Hannover Medical School, Germany

^3^ Department of Pediatric Pulmology, Hannover Medical School, Germany

***Correspondence should be adressed to:**

Ivonne Wieland, MD

Department of Pediatric Hematology and Oncology,

Hannover Medical School, Germany

Carl-Neuberg-Str. 1, 30625 Hannover

Email: wieland.ivonne@mh-hannover.de

or

Georg Hansmann, MD, PhD

Department of Pediatric Cardiology and Critrical Care

Hannover Medical School, Germany

Carl-Neuberg-Str. 1, 30625 Hannover

Email: georg.hansmann@gmail.com

**Keywords**

Acquired von Willebrand Syndrome, platelet function, pulmonary arterial hypertension, pulmonary hypertension, children

**Table S1. Individual patient characteristics and medication before lung transplantation**

| **ID** | **Age (years)** | **Gender**  **(M/F)** | **Weight (kg)** | **BSA (m²)** | **FC**  **(1-4)** | **NTproBNP**  **(pg/mL)** | **Diagnosis** | **PH relevant medication pre LuTx** |
| --- | --- | --- | --- | --- | --- | --- | --- | --- |
| **1** | 15.0 | F | 43.0 (4^th^ Perc.) | 1.40 | 4 | 5203 | PCH/PAH  (group 1.6 PH) | ILO, SIL, BOS, SPI, FUR, 15 L O_2_/min |
| **2** | 13.2 | F | 45.0 (31^st^ Perc.) | 1.50 | 4 | 4436 | IPAH  (group 1.1 PH),  type 1 diabetes | EPIV, SIL, BOS, FUR |
| **3** | 10.7 | M | 35.0  (44^th^ Perc.) | 1.30 | 4 | 1482 | IPAH  (group 1.1 PH) | TREP i.v., SIL, MAC, SPI, ASA, 2-3 L O_2_/min |
| **4** | 14.2 | F | 47.0  (23^rd^ Perc.) | 1.50 | 4 | 10972 | PAH-CHD  (group 1.4.4 PH)  s/p d-TGA repair | TREP i.v., RIO, BOS, 2 L O_2_/min |
| **5** | 1.9 | M | 8.2  (< 1^st^ Perc.) | 0.42 | 3-4 | 8384 | PAH-CHD (group 1.4.4 PH), Preterm 29+2 GW, IRDS, prothrombin mutation, severe RPA hypoplasia, ASD II, s/p PDA closure | SIL, MAC, CLO, SPI, 0.75 L O_2_/min |
| **6** | 2.3 | F | 8.9  (< 1^st^ Perc.) | 0.43 | 4 | 9720 | IPAH  (group 1.1 PH),  ASD II | ILO inhal., SIL, MAC, SEL, SPI, 2-3 L O_2_/min |
| **7** | 17.5 | F | 39.0  (< 1^st^ Perc.) | 1.30 | 3 | 743 | HPAH, BMPR2 mutation  (group 1.2 PH),  M. Osler (HHT), small PFO | ILO inhal., SIL, MAC, SPI |
| **8** | 10.3 | F | 25.0  (3^rd^ Perc.) | 0.91 | 4 | 110 | HPAH, TBX4 mutation  (group 1.2 PH),  IRDS, chILD, PFO, small patella syndrome | ILO inhal., SIL, MAC, SPI, 1.5-2.5 L O_2_/min |
| **9** | 11.7 | M | 32.0  (11^th^ Perc.) | 1.10 | 4 | 7596 | PVOD/PAH  (group 1.6 PH),  Preterm 32+5 GW, s/p gastroschisis, double aortic arch with atresia of the left arch, type 2 vWD, intervent. rASD 09/2017 | TREP i.v., TAD, BOS, AML, SPI, ASA |
| **10** | 17.8 | F | 57.0  (42^nd^ Perc.) | 1.70 | 3 | 566 | IPAH  (group 1.1 PH),  type 2 vWD, migraine | TREP i.v. infusion pump, SIL, MAC, DIG, SPI |
| **11** | 16.2 | F | 51.0  (20^th^ Perc.) | 1.50 | 4 | 444 | IPAH  (group 1.1 PH), intervent. rASD 01/2019 | ILO inhal., SIL, MAC, AML, ASA, SPI, 2L O_2_/min |
| **12** | 11.1 | F | 40.0  (59^th^ Perc.) | 1.30 | 4 | 243 | PVOD/PCH/PAH with plexiform lesions (group 1.6 PH), partial anomalous pulmonary venous connection | Levosimendan, Milrinone, SIL, MAC |
| **13** | 5.5 | F | 16.8  (9^th^ Perc.) | 0.74 | 3 | 359 | HPAH, BMPR2 mutation  (group 1.2 PH) | EPIV, SIL, BOS, AML |
| **14** | 8.2 | F | 22.2  (9^th^ Perc.) | 0.91 | 3-4 | 273 | HPAH, BMPR2 mutation  (group 1.2 PH), s/p VSD repair | Levosimendan (repetitive), SIL, MAC, SEL, SPI |
| **15** | 6.3 | F | 14.7  (< 1^st^ Perc.) | 0.66 | 3 | 408 | IPAH, heterozygous EIF2AK4 mutation of unclear significance (group 1.1 PH), ASD II | RIO, MAC, SEL, SPI |
| **16** | 21.3 | F | 63.0 | 1.86 | 3 | 3448 | PAH-CHD  (group 1.4.4 PH) repaired AP window | Levosimendan (repetitive), TREP i.v. infusion pump, RIO, MAC |
| **17** | 3.2 | F | 10.0  (<1^st^ Perc.) | 0.49 | 4 | 5889 | HPAH, BMPR2 mutation (group 1.2 PH); Preterm 34+4 GW, CPAM type 1, s/p resection of the left lower lobe and lingula, PPHN | Levosimendan (repetitive), TAD, MAC, SPI, High Flow FiO2 25% |
| **18** | 6.8 | F | 20.0  (16^th^ Perc.) | 0.85 | 4 | 491 | IPAH  (group 1.1 PH) | EPIV, SIL, AML, inhaled NO |
| **19** | 15.5 | F | 48.0  (13^th^ Perc.) | 1.48 | 3 | 275 | IPAH (group 1.1 PH) or PAH-CHD (group 1.4.4 PH); Preterm 27 + 0 GW, BPD, large PDA with right to left shunt, asthma | TREP i.v. infusion pump, SIL, MAC, |
| **20** | 8.7 | M | 21.0 (1^st^ Perc.) | 0.92 | 3 | 276 | PH associated with interstitial lung disease (group 3 PH) chILD, small ASD, PEG | ILO inhal., RIO, MAC, SEL, SPI, ASA |

# Table S2. Individual PH patient characteristics and medication before lung transplantation. The serum N-terminal prohormone of brain natriuretic peptide (NTproBNP) concentrations are the last measurements prior to LuTx. If the patient has a mutation that is associated with PAH, he/she belongs to group 1.2 PH (HPAH). Abbreviations: AML, amlodipine; ASA, acetylsalicylic acid (P.O.); ASD, atrial septal defect; BSA, body surface area; BPD, bronchopulmonary dysplasia; CHD, congenital heart disease; CLO, clopidogrel; CPAM, congenital pulmonary airway malformation; CPB, cardiopulmonary bypass; CPR, cardiopulmonary resuscitation; digoxin (P.O.); EPIV; epoprostenol i.v.; FUR, furosemide (P.O.); HPAH, hereditary PAH; ILO, iloprost; IPAH, idiopathic PAH; intervent. rASD, interventional creation of a restrictive atrial septal defect; LuTx, lung transplantation; NT-proBNP, N-terminal pro b-type natriuretic peptide; PAH, pulmonary arterial hypertension; PCH, pulmonary capillary hemangiomatosis; PDA, patent ductus arteriosus; PVOD, pulmonary venoocclusive disease; SEL, selexipag (P.O.); SIL, sildenafil (P.O.); SPI, spironolactone (P.O.); TAD, Tadalafil; TREP, treprostinil; VA-ECMO, veno-arterial extracorporeal membrane oxygenation
